# *tert*-Butyl *N*-[2-(*N*-isobutyl-4-meth­oxy­benzene­sulfonamido)­eth­yl]carbamate

**DOI:** 10.1107/S1600536814009143

**Published:** 2014-05-17

**Authors:** Xiao-Guang Bai, Ju-Xian Wang

**Affiliations:** a Institute of Medicinal Biotechnology, Chinese Academy of Medical Sciences and Peking Union Medical College, Beijing 100050, People’s Republic of China

## Abstract

The title compound, C_18_H_30_N_2_O_5_S, was synthesized by the reaction of *tert*-butyl 2-(iso­butyl­amino)­ethyl­carbamate with *p*-meth­oxy­phenyl­sulfonyl chloride. In the mol­ecule, two intra­molecular C—H⋯O hydrogen bonds are observed. In the crystal, mol­ecules are linked by N—H⋯O hydrogen bonds involving the imino group N atom and the ester group O atom into chains running parallel to the *b* axis. The chains are further connected by C—H⋯O hydrogen bonds, forming layers parallel to the *bc* plane.

## Related literature   

For potential HIV-1 protease inhibitors, see: Surleraux *et al.* (2005 ▶); Ghosh *et al.* (2006 ▶, 2011 ▶); Guo *et al.* (2010 ▶). For the structure of the meth­oxy analogue, see: Chatziefthimiou *et al.* (2006 ▶)
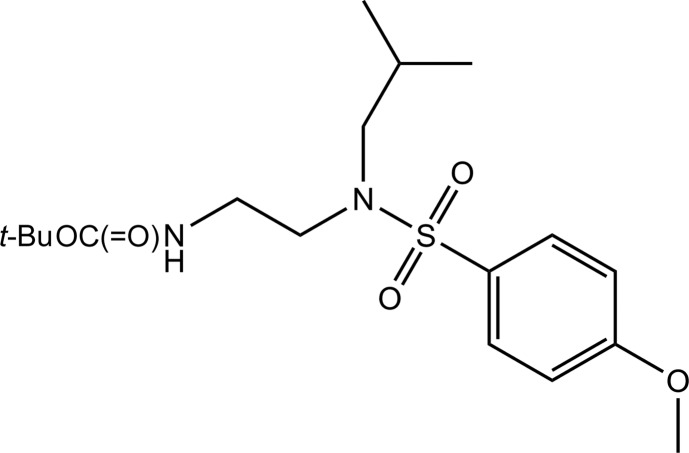


## Experimental   

### 

#### Crystal data  

C_18_H_30_N_2_O_5_S
*M* *_r_* = 386.50
Monoclinic, 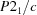

*a* = 19.2484 (5) Å
*b* = 5.29088 (12) Å
*c* = 20.1825 (6) Å
β = 92.497 (3)°
*V* = 2053.46 (9) Å^3^
*Z* = 4
Cu *K*α radiation
μ = 1.65 mm^−1^
*T* = 293 K
0.28 × 0.16 × 0.14 mm

#### Data collection  

Agilent Xcalibur (Atlas, Gemini ultra) diffractometer
Absorption correction: multi-scan (*CrysAlis PRO*; Agilent, 2013 ▶) *T*_min_ = 0.776, *T*_max_ = 1.000
11865 measured reflections
3658 independent reflections
3122 reflections with *I* > 2σ(*I*)
*R*_int_ = 0.031

#### Refinement  

*R*[*F* ^2^ > 2σ(*F* ^2^)] = 0.034
*wR*(*F* ^2^) = 0.089
*S* = 1.05
3658 reflections
245 parameters
H atoms treated by a mixture of independent and constrained refinement
Δρ_max_ = 0.17 e Å^−3^
Δρ_min_ = −0.30 e Å^−3^

### 

Data collection: *CrysAlis PRO* (Agilent, 2013 ▶); cell refinement: *CrysAlis PRO*; data reduction: *CrysAlis PRO*; program(s) used to solve structure: *SHELXS97* (Sheldrick, 2008 ▶); program(s) used to refine structure: *SHELXL97* (Sheldrick, 2008 ▶); molecular graphics: *SHELXTL/PC* (Sheldrick, 2008 ▶); software used to prepare material for publication: *SHELXTL/PC*.

## Supplementary Material

Crystal structure: contains datablock(s) I, New_Global_Publ_Block. DOI: 10.1107/S1600536814009143/rz5120sup1.cif

Structure factors: contains datablock(s) I. DOI: 10.1107/S1600536814009143/rz5120Isup2.hkl

Supporting information file. DOI: 10.1107/S1600536814009143/rz5120Isup3.cml

CCDC reference: 998938

Additional supporting information:  crystallographic information; 3D view; checkCIF report

## Figures and Tables

**Table 1:** Hydrogen-bond geometry (Å, °)

*D*—H⋯*A*	*D*—H	H⋯*A*	*D*⋯*A*	*D*—H⋯*A*
C8—H8*A*⋯O2	0.97	2.43	2.9106 (19)	110
C13—H13*A*⋯O3	0.97	2.48	3.107 (2)	122
C3—H3⋯O4^i^	0.93	2.59	3.402 (2)	147
N2—H2*A*⋯O4^ii^	0.82 (2)	2.38 (2)	3.190 (2)	171 (2)

Symmetry codes: (i) 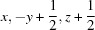; (ii) 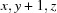.

**Fig. 1.**
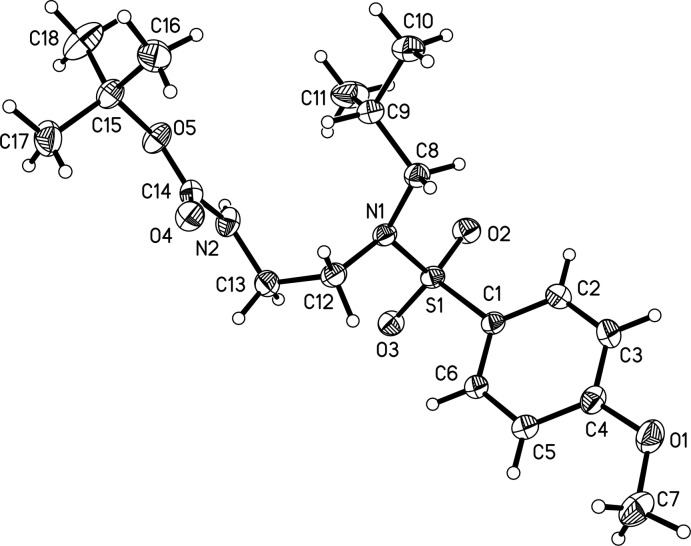
The molecular structure of the title compound, showing displacement ellipsoids drawn at the 30% probability level.

**Fig. 2.**
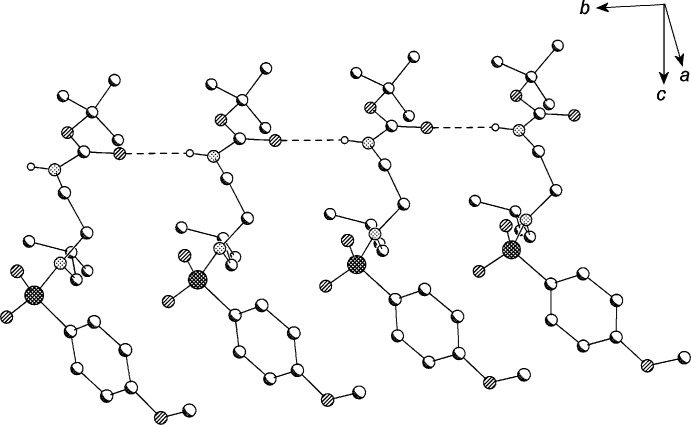
Partial packing diagram of the title compound showing the formation of a molecular chain through N—H···O hydrogen bonds. Hydrogen atoms not involved in hydrogen bonding (dashed lines) are omitted.

## supplementary crystallographic information

### 1. Comment

As a part of our ongoing project aimed at the development of potential HIV-1 protease inhibitors (Surleraux *et al.*, 2005; Ghosh *et al.*, 2006, 2011; Guo *et al.*, 2010), we have synthesized the title compound and report its crystal structure herein.

The molecular structure of the title compound is illustrated in Fig. 1. Bond distances and angles are similar to those found in the methoxy analogue (Chatziefthimiou *et al.*, 2006). The molecular conformation is stabilized by two intramolecular C—H···O hydrogen bonds (Table 1). In the crystal, the molecules are linked into chains by intermolecular N—H···O hydrogen bonds (Table 1) parallel to the *b* axis (Fig. 2), which are further connected to form layers parallel to the *bc* plane by C—H···O hydrogen bonds (Table 1).

### 2. Experimental

To a solution of *tert*-butyl 2-(isobutylamino)ethylcarbamate (1.13 g, 5.2 mmol) and "*N,N*-diisopropylethylamine (1.34 g, 10.4 mmol) in dichloromethane (10 ml) was added dropwise a solution of *p*-methoxyphenylsulfonyl chloride (1.18 g, 5.7 mmol) in dichloromethane (3 ml) over a period of 10 min at room temperature. The reaction mixture was stirred for 5 h at the same temperature and concentrated under reduced pressure. t*ert*-Butyl 2-(*N*-isobutyl-4-methoxyphenylsulfonamido)ethylcarbamate was obtained as a white solid by flash chromatography (40 g silica gel, petroleum ether/AcOEt, 1:10 *v*/*v*). The yield is 42%. Colourless block crystals suitable for X-ray diffraction were obtained in 3 day by slow evaporation of a petroleum ether/AcOEt (4:1 *v*/*v*) solution.

### 3. Refinement

All H atoms could be detected in a difference Fourier map. The H atom bonded to N2 was refined freely, all other Hatoms were placed in calculated positions and refined using a riding motion approxmation, with C–H = 0.93–0.97 Å, and with *U*_iso_(H) = 1.2*U*_eq_(C) or 1.5*U*_eq_(C) for methyl H atoms.

### Crystal data

**Table:**

C_18_H_30_N_2_O_5_S	*F*(000) = 832
*M**_r_* = 386.50	*D*_x_ = 1.250 Mg m^−^^3^
Monoclinic, *P*2_1_/*c*	Cu *K*α radiation, λ = 1.54184 Å
Hall symbol: -P 2ybc	Cell parameters from 5271 reflections
*a* = 19.2484 (5) Å	θ = 4.4–67.1°
*b* = 5.29088 (12) Å	µ = 1.65 mm^−^^1^
*c* = 20.1825 (6) Å	*T* = 293 K
β = 92.497 (3)°	Block, colorless
*V* = 2053.46 (9) Å^3^	0.28 × 0.16 × 0.14 mm
*Z* = 4	

### Data collection

**Table:**

Agilent Xcalibur (Atlas, Gemini ultra) diffractometer	3658 independent reflections
Radiation source: Enhance Ultra (Cu) X-ray Source	3122 reflections with *I* > 2σ(*I*)
Mirror monochromator	*R*_int_ = 0.031
Detector resolution: 10.4713 pixels mm^-1^	θ_max_ = 67.2°, θ_min_ = 4.4°
ω scans	*h* = −21→22
Absorption correction: multi-scan (*CrysAlis PRO*; Agilent, 2013)	*k* = −4→6
*T*_min_ = 0.776, *T*_max_ = 1.000	*l* = −22→24
11865 measured reflections	

### Refinement

**Table:**

Refinement on *F*^2^	Primary atom site location: structure-invariant direct methods
Least-squares matrix: full	Secondary atom site location: difference Fourier map
*R*[*F*^2^ > 2σ(*F*^2^)] = 0.034	Hydrogen site location: inferred from neighbouring sites
*wR*(*F*^2^) = 0.089	H atoms treated by a mixture of independent and constrained refinement
*S* = 1.05	*w* = 1/[σ^2^(*F*_o_^2^) + (0.0429*P*)^2^ + 0.3785*P*] where *P* = (*F*_o_^2^ + 2*F*_c_^2^)/3
3658 reflections	(Δ/σ)_max_ = 0.001
245 parameters	Δρ_max_ = 0.17 e Å^−^^3^
0 restraints	Δρ_min_ = −0.30 e Å^−^^3^

### Special details

**Table:**

Geometry. All e.s.d.'s (except the e.s.d. in the dihedral angle between two l.s. planes) are estimated using the full covariance matrix. The cell e.s.d.'s are taken into account individually in the estimation of e.s.d.'s in distances, angles and torsion angles; correlations between e.s.d.'s in cell parameters are only used when they are defined by crystal symmetry. An approximate (isotropic) treatment of cell e.s.d.'s is used for estimating e.s.d.'s involving l.s. planes.
Refinement. Refinement of *F*^2^ against ALL reflections. The weighted *R*-factor *wR* and goodness of fit *S* are based on *F*^2^, conventional *R*-factors *R* are based on *F*, with *F* set to zero for negative *F*^2^. The threshold expression of *F*^2^ > σ(*F*^2^) is used only for calculating *R*-factors(gt) *etc*. and is not relevant to the choice of reflections for refinement. *R*-factors based on *F*^2^ are statistically about twice as large as those based on *F*, and *R*- factors based on ALL data will be even larger.

### Fractional atomic coordinates and isotropic or equivalent isotropic displacement parameters (Å^2^)

**Table:**

	*x*	*y*	*z*	*U*_iso_*/*U*_eq_	
C1	0.85965 (7)	0.6201 (3)	0.54057 (7)	0.0368 (3)	
C2	0.82937 (9)	0.6078 (3)	0.60169 (8)	0.0474 (4)	
H2	0.7921	0.7121	0.6107	0.057*	
C3	0.85494 (9)	0.4403 (4)	0.64878 (8)	0.0527 (4)	
H3	0.8347	0.4314	0.6897	0.063*	
C4	0.91066 (8)	0.2845 (3)	0.63579 (8)	0.0440 (4)	
C5	0.94134 (8)	0.2961 (3)	0.57509 (8)	0.0435 (4)	
H5	0.9788	0.1921	0.5662	0.052*	
C6	0.91528 (8)	0.4657 (3)	0.52769 (7)	0.0403 (3)	
H6	0.9356	0.4754	0.4868	0.048*	
C7	0.98789 (12)	−0.0418 (4)	0.67445 (11)	0.0687 (6)	
H7A	0.9749	−0.1530	0.6384	0.103*	
H7B	0.9983	−0.1396	0.7137	0.103*	
H7C	1.0282	0.0537	0.6634	0.103*	
C8	0.70575 (8)	0.5843 (3)	0.45894 (8)	0.0420 (3)	
H8A	0.6984	0.6863	0.4980	0.050*	
H8B	0.7095	0.4093	0.4730	0.050*	
C9	0.64333 (8)	0.6121 (3)	0.41093 (8)	0.0447 (4)	
H9	0.6499	0.5001	0.3730	0.054*	
C10	0.57790 (9)	0.5294 (4)	0.44490 (10)	0.0602 (5)	
H10A	0.5853	0.3658	0.4645	0.090*	
H10B	0.5397	0.5213	0.4128	0.090*	
H10C	0.5675	0.6491	0.4788	0.090*	
C11	0.63562 (11)	0.8797 (4)	0.38541 (12)	0.0743 (6)	
H11A	0.6288	0.9921	0.4219	0.111*	
H11B	0.5963	0.8894	0.3546	0.111*	
H11C	0.6769	0.9276	0.3635	0.111*	
C12	0.80312 (8)	0.4772 (3)	0.38535 (8)	0.0426 (3)	
H12A	0.7712	0.3368	0.3783	0.051*	
H12B	0.8454	0.4114	0.4069	0.051*	
C13	0.82046 (9)	0.5869 (4)	0.31821 (8)	0.0517 (4)	
H13A	0.8475	0.7399	0.3254	0.062*	
H13B	0.8493	0.4669	0.2956	0.062*	
C14	0.72163 (8)	0.4609 (3)	0.24696 (7)	0.0410 (3)	
C15	0.62067 (9)	0.3966 (3)	0.17124 (8)	0.0459 (4)	
C16	0.57907 (11)	0.2465 (4)	0.21953 (10)	0.0642 (5)	
H16A	0.6083	0.1208	0.2407	0.096*	
H16B	0.5408	0.1655	0.1961	0.096*	
H16C	0.5617	0.3581	0.2525	0.096*	
C17	0.65827 (11)	0.2296 (4)	0.12362 (9)	0.0646 (5)	
H17A	0.6895	0.3305	0.0989	0.097*	
H17B	0.6250	0.1496	0.0936	0.097*	
H17C	0.6842	0.1028	0.1481	0.097*	
C18	0.57448 (13)	0.5846 (4)	0.13381 (13)	0.0827 (7)	
H18A	0.5534	0.6958	0.1647	0.124*	
H18B	0.5388	0.4956	0.1086	0.124*	
H18C	0.6019	0.6816	0.1044	0.124*	
N1	0.77172 (6)	0.6617 (2)	0.42986 (6)	0.0371 (3)	
N2	0.76011 (8)	0.6456 (3)	0.27567 (7)	0.0474 (3)	
O1	0.93204 (7)	0.1260 (3)	0.68599 (6)	0.0611 (3)	
O2	0.78598 (6)	1.0150 (2)	0.50924 (6)	0.0505 (3)	
O3	0.88216 (6)	0.8952 (2)	0.43790 (5)	0.0462 (3)	
O4	0.73211 (6)	0.2357 (2)	0.25588 (6)	0.0495 (3)	
O5	0.67038 (6)	0.5608 (2)	0.20784 (6)	0.0511 (3)	
S1	0.825787 (18)	0.82405 (6)	0.478139 (18)	0.03723 (12)	
H2A	0.7503 (11)	0.793 (4)	0.2665 (10)	0.060 (6)*	

### Atomic displacement parameters (Å^2^)

**Table:**

	*U*^11^	*U*^22^	*U*^33^	*U*^12^	*U*^13^	*U*^23^
C1	0.0318 (7)	0.0407 (7)	0.0375 (7)	−0.0033 (6)	−0.0025 (6)	−0.0012 (6)
C2	0.0405 (8)	0.0577 (10)	0.0443 (8)	0.0031 (7)	0.0057 (7)	−0.0026 (7)
C3	0.0501 (10)	0.0697 (11)	0.0390 (8)	−0.0005 (8)	0.0101 (7)	0.0035 (8)
C4	0.0441 (8)	0.0474 (8)	0.0400 (8)	−0.0088 (7)	−0.0052 (6)	0.0062 (7)
C5	0.0372 (8)	0.0486 (9)	0.0442 (8)	0.0021 (7)	−0.0017 (6)	0.0011 (7)
C6	0.0345 (7)	0.0497 (8)	0.0366 (7)	0.0005 (6)	0.0007 (6)	0.0017 (6)
C7	0.0723 (13)	0.0631 (12)	0.0693 (12)	0.0052 (10)	−0.0139 (10)	0.0211 (10)
C8	0.0367 (8)	0.0466 (8)	0.0428 (8)	−0.0027 (7)	0.0007 (6)	0.0046 (7)
C9	0.0363 (8)	0.0500 (9)	0.0476 (8)	0.0010 (7)	−0.0013 (6)	0.0004 (7)
C10	0.0389 (9)	0.0742 (12)	0.0675 (11)	−0.0056 (9)	0.0011 (8)	0.0022 (10)
C11	0.0536 (11)	0.0713 (13)	0.0980 (16)	0.0155 (10)	0.0030 (11)	0.0339 (12)
C12	0.0400 (8)	0.0428 (8)	0.0444 (8)	0.0040 (7)	−0.0050 (6)	−0.0050 (7)
C13	0.0422 (9)	0.0688 (11)	0.0442 (8)	−0.0069 (8)	0.0024 (7)	−0.0105 (8)
C14	0.0461 (8)	0.0429 (8)	0.0342 (7)	−0.0037 (7)	0.0034 (6)	−0.0027 (6)
C15	0.0509 (9)	0.0383 (8)	0.0475 (8)	−0.0074 (7)	−0.0082 (7)	−0.0010 (7)
C16	0.0576 (11)	0.0712 (12)	0.0645 (11)	−0.0108 (10)	0.0085 (9)	0.0014 (10)
C17	0.0709 (13)	0.0721 (12)	0.0509 (10)	−0.0149 (10)	0.0038 (9)	−0.0163 (9)
C18	0.0884 (16)	0.0549 (11)	0.1003 (17)	−0.0064 (11)	−0.0478 (14)	0.0101 (11)
N1	0.0324 (6)	0.0399 (6)	0.0386 (6)	−0.0009 (5)	−0.0021 (5)	−0.0010 (5)
N2	0.0557 (8)	0.0455 (8)	0.0405 (7)	−0.0096 (7)	−0.0031 (6)	−0.0014 (6)
O1	0.0663 (8)	0.0678 (8)	0.0489 (7)	0.0010 (7)	−0.0025 (6)	0.0188 (6)
O2	0.0520 (7)	0.0400 (6)	0.0593 (7)	0.0084 (5)	−0.0007 (5)	−0.0097 (5)
O3	0.0422 (6)	0.0462 (6)	0.0502 (6)	−0.0078 (5)	0.0016 (5)	0.0059 (5)
O4	0.0555 (7)	0.0411 (6)	0.0512 (6)	0.0035 (5)	−0.0035 (5)	−0.0034 (5)
O5	0.0606 (7)	0.0367 (6)	0.0543 (6)	−0.0050 (5)	−0.0165 (5)	−0.0016 (5)
S1	0.03497 (19)	0.03481 (19)	0.0416 (2)	−0.00052 (14)	−0.00156 (14)	−0.00101 (14)

### Geometric parameters (Å, º)

**Table:**

C1—C6	1.381 (2)	C11—H11C	0.9600
C1—C2	1.389 (2)	C12—N1	1.474 (2)
C1—S1	1.7621 (15)	C12—C13	1.524 (2)
C2—C3	1.375 (2)	C12—H12A	0.9700
C2—H2	0.9300	C12—H12B	0.9700
C3—C4	1.387 (3)	C13—N2	1.448 (2)
C3—H3	0.9300	C13—H13A	0.9700
C4—O1	1.365 (2)	C13—H13B	0.9700
C4—C5	1.384 (2)	C14—O4	1.2205 (19)
C5—C6	1.389 (2)	C14—N2	1.342 (2)
C5—H5	0.9300	C14—O5	1.345 (2)
C6—H6	0.9300	C15—O5	1.4677 (19)
C7—O1	1.421 (3)	C15—C17	1.513 (3)
C7—H7A	0.9600	C15—C16	1.513 (3)
C7—H7B	0.9600	C15—C18	1.514 (3)
C7—H7C	0.9600	C16—H16A	0.9600
C8—N1	1.4797 (19)	C16—H16B	0.9600
C8—C9	1.518 (2)	C16—H16C	0.9600
C8—H8A	0.9700	C17—H17A	0.9600
C8—H8B	0.9700	C17—H17B	0.9600
C9—C11	1.512 (3)	C17—H17C	0.9600
C9—C10	1.524 (2)	C18—H18A	0.9600
C9—H9	0.9800	C18—H18B	0.9600
C10—H10A	0.9600	C18—H18C	0.9600
C10—H10B	0.9600	N1—S1	1.6378 (12)
C10—H10C	0.9600	N2—H2A	0.82 (2)
C11—H11A	0.9600	O2—S1	1.4294 (12)
C11—H11B	0.9600	O3—S1	1.4338 (12)
			
C6—C1—C2	119.91 (14)	N1—C12—H12B	108.8
C6—C1—S1	119.56 (11)	C13—C12—H12B	108.8
C2—C1—S1	120.50 (12)	H12A—C12—H12B	107.7
C3—C2—C1	119.52 (15)	N2—C13—C12	114.06 (14)
C3—C2—H2	120.2	N2—C13—H13A	108.7
C1—C2—H2	120.2	C12—C13—H13A	108.7
C2—C3—C4	120.60 (15)	N2—C13—H13B	108.7
C2—C3—H3	119.7	C12—C13—H13B	108.7
C4—C3—H3	119.7	H13A—C13—H13B	107.6
O1—C4—C5	123.89 (16)	O4—C14—N2	124.23 (15)
O1—C4—C3	115.82 (15)	O4—C14—O5	125.63 (14)
C5—C4—C3	120.29 (15)	N2—C14—O5	110.14 (14)
C4—C5—C6	118.87 (15)	O5—C15—C17	110.24 (14)
C4—C5—H5	120.6	O5—C15—C16	109.76 (14)
C6—C5—H5	120.6	C17—C15—C16	112.57 (16)
C1—C6—C5	120.81 (14)	O5—C15—C18	102.60 (13)
C1—C6—H6	119.6	C17—C15—C18	110.70 (17)
C5—C6—H6	119.6	C16—C15—C18	110.52 (18)
O1—C7—H7A	109.5	C15—C16—H16A	109.5
O1—C7—H7B	109.5	C15—C16—H16B	109.5
H7A—C7—H7B	109.5	H16A—C16—H16B	109.5
O1—C7—H7C	109.5	C15—C16—H16C	109.5
H7A—C7—H7C	109.5	H16A—C16—H16C	109.5
H7B—C7—H7C	109.5	H16B—C16—H16C	109.5
N1—C8—C9	112.90 (12)	C15—C17—H17A	109.5
N1—C8—H8A	109.0	C15—C17—H17B	109.5
C9—C8—H8A	109.0	H17A—C17—H17B	109.5
N1—C8—H8B	109.0	C15—C17—H17C	109.5
C9—C8—H8B	109.0	H17A—C17—H17C	109.5
H8A—C8—H8B	107.8	H17B—C17—H17C	109.5
C11—C9—C8	111.82 (15)	C15—C18—H18A	109.5
C11—C9—C10	110.57 (16)	C15—C18—H18B	109.5
C8—C9—C10	109.38 (13)	H18A—C18—H18B	109.5
C11—C9—H9	108.3	C15—C18—H18C	109.5
C8—C9—H9	108.3	H18A—C18—H18C	109.5
C10—C9—H9	108.3	H18B—C18—H18C	109.5
C9—C10—H10A	109.5	C12—N1—C8	116.13 (12)
C9—C10—H10B	109.5	C12—N1—S1	116.31 (10)
H10A—C10—H10B	109.5	C8—N1—S1	116.30 (10)
C9—C10—H10C	109.5	C14—N2—C13	120.87 (16)
H10A—C10—H10C	109.5	C14—N2—H2A	118.5 (15)
H10B—C10—H10C	109.5	C13—N2—H2A	120.5 (15)
C9—C11—H11A	109.5	C4—O1—C7	117.91 (14)
C9—C11—H11B	109.5	C14—O5—C15	120.56 (12)
H11A—C11—H11B	109.5	O2—S1—O3	119.77 (7)
C9—C11—H11C	109.5	O2—S1—N1	107.05 (7)
H11A—C11—H11C	109.5	O3—S1—N1	106.12 (7)
H11B—C11—H11C	109.5	O2—S1—C1	107.89 (7)
N1—C12—C13	113.64 (13)	O3—S1—C1	107.48 (7)
N1—C12—H12A	108.8	N1—S1—C1	108.05 (7)
C13—C12—H12A	108.8		
			
C6—C1—C2—C3	−0.4 (2)	C5—C4—O1—C7	−1.8 (2)
S1—C1—C2—C3	177.48 (13)	C3—C4—O1—C7	178.47 (16)
C1—C2—C3—C4	0.1 (3)	O4—C14—O5—C15	0.1 (2)
C2—C3—C4—O1	179.80 (16)	N2—C14—O5—C15	−179.50 (14)
C2—C3—C4—C5	0.1 (3)	C17—C15—O5—C14	−61.01 (19)
O1—C4—C5—C6	−179.78 (15)	C16—C15—O5—C14	63.5 (2)
C3—C4—C5—C6	−0.1 (2)	C18—C15—O5—C14	−178.95 (17)
C2—C1—C6—C5	0.4 (2)	C12—N1—S1—O2	171.49 (10)
S1—C1—C6—C5	−177.50 (12)	C8—N1—S1—O2	−46.10 (12)
C4—C5—C6—C1	−0.1 (2)	C12—N1—S1—O3	42.49 (12)
N1—C8—C9—C11	−57.0 (2)	C8—N1—S1—O3	−175.09 (10)
N1—C8—C9—C10	−179.83 (14)	C12—N1—S1—C1	−72.54 (12)
N1—C12—C13—N2	−70.15 (18)	C8—N1—S1—C1	69.88 (12)
C13—C12—N1—C8	127.99 (14)	C6—C1—S1—O2	−161.35 (12)
C13—C12—N1—S1	−89.53 (14)	C2—C1—S1—O2	20.79 (15)
C9—C8—N1—C12	−78.70 (17)	C6—C1—S1—O3	−30.90 (14)
C9—C8—N1—S1	138.82 (12)	C2—C1—S1—O3	151.23 (13)
O4—C14—N2—C13	2.9 (2)	C6—C1—S1—N1	83.23 (13)
O5—C14—N2—C13	−177.48 (14)	C2—C1—S1—N1	−94.64 (13)
C12—C13—N2—C14	−72.1 (2)		

### Hydrogen-bond geometry (Å, º)

**Table:**

*D*—H···*A*	*D*—H	H···*A*	*D*···*A*	*D*—H···*A*
C8—H8*A*···O2	0.97	2.43	2.9106 (19)	110
C13—H13*A*···O3	0.97	2.48	3.107 (2)	122
C3—H3···O4^i^	0.93	2.59	3.402 (2)	147
N2—H2*A*···O4^ii^	0.82 (2)	2.38 (2)	3.190 (2)	171 (2)

Symmetry codes: (i) *x*, −*y*+1/2, *z*+1/2; (ii) *x*, *y*+1, *z*.
